# Nonpneumatic anti-shock garment utilization for obstetric hemorrhage management and its predictors among obstetric care providers in Ethiopia: a systematic review and meta-analysis

**DOI:** 10.1186/s12913-024-11333-0

**Published:** 2024-08-01

**Authors:** Eyob Shitie Lake, Mulat Ayele, Gizachew Yilak, Befkad Derese Tilahun, Besfat Berihun Erega, Alemu Birara Zemariam, Getinet Kumie

**Affiliations:** 1 Department of Midwifery, College of Medicine and Health Science, Woldia University, Woldia, Ethiopia; 2 Department of Nursing, College of Medicine and Health Science, Woldia University, Woldia, Ethiopia; 3 Department of Midwifery, College of Medicine and Health Science, Debre Tabor University, Debre Tabor, Ethiopia; 4 Department of Medical Laboratory, College of Medicine and Health Science, Woldia University, Woldia, Ethiopia

**Keywords:** NASG, Obstetric hemorrhage, Ethiopia

## Abstract

**Introduction:**

Non-pneumatic Anti-Shock Garment (NASG) is a lightweight, reusable first aid compression device that squeezes blood from the lower extremities and centralizes blood circulation to vital organs of the body. Postpartum hemorrhage followed by severe preeclampsia/eclampsia is the leading primary cause of maternal death (A reduction in extreme maternal adverse outcomes and faster recovery from shock are more likely to occur with earlier NASG intervention. The median blood loss reduced by half when the NASG was used for obstetric hemorrhage management, which was associated with significantly reduced maternal mortality among the most severe cases.

**Objective:**

To estimate the pooled prevalence of NASG utilization and its predictors in Ethiopia.

**Methods:**

Appropriate and comprehensive searches of PubMed, MEDLINE, EMBASE, Google Scholar, HINARI, and Scopus were performed. The electronic literature search was last performed on November 18/2023. All observational study designs were eligible for this SRMA. All cross sectional studies reporting the prevalence/proportion of NASG utilization for obstetric hemorrhage management among obstetric care providers and associated factors were included in this SRMA. Primary studies lacking the outcome of interest were excluded from the SRMA. The extracted Microsoft Excel spreadsheet data were imported into STATA software version 17 (STATA Corporation, Texas, USA) for analysis. A random-effects model was used to estimate the pooled prevalence of NASG utilization among obstetric care providers in Ethiopia. The Cochrane Q-test and I^2^ statistics were computed to assess the heterogeneity among the studies included in the SRMA.

**Result:**

A total of 1623 articles were found by using our search strategies and seven studies comprising 2335 participants were ultimately included in the SRMA. The pooled prevalence of NASG utilization for obstetric hemorrhage in Ethiopia was 43.34% (95% CI: 35.25, 51.42%). The findings of this subgroup analysis by sample size showed that the pooled prevalence of NASG utilization for obstetric hemorrhage was greater in studies with sample sizes of less than the mean sample size (48.6%; 95% CI: 32.34, 64.86%). Receiving training (AOR = 3.88, 95% CI: 2.08–5.37), having good knowledge (AOR = 1.99, 95% CI: 1.28–3.16), positive attitude (AOR = 2.16, 95% CI: 1.62–2.75) and having available NASGs in the facility (AOR = 4.89, 95%CI: 2.88–8.32) were significantly associated with the use of NASGs for obstetric hemorrhage management.

**Conclusion:**

The level of NASG utilization for obstetric hemorrhage in Ethiopia is low. Receiving training, good knowledge, positive attitudes and availability of NASG were significantly associated with the utilization of NASG. Therefore, policy makers and other stakeholders should emphasize enhancing the knowledge and attitudes of obstetric care providers through continuous support and training. At the same time, they should work strictly in providing devices for all the health facilities.

**Supplementary Information:**

The online version contains supplementary material available at 10.1186/s12913-024-11333-0.

## Introduction

Non-Pneumatic Anti-Shock Garment (NASG) is a lightweight (1.5 kg) reusable first aid compression device that squeezes blood from the lower extremities and centralizes blood circulation to vital organs of the body. With minimal training, health care providers can successfully treat patients with bleeding, which can reverse hypovolemic shock resulting from obstetric hemorrhage. Once the mother’s bleeding is controlled, she can be safely transported to a hospital for obstetric care [1]. Globally, approximately 800 women die every day from preventable causes related to pregnancy and childbirth [2]. Although the maternal mortality ratio has decreased by approximately 38% worldwide, 94% of all maternal deaths occur in low and lower middle-income countries peaked in sub-Saharan Africa with 510 deaths per 100,000 live births [3]. According to the Ethiopian demographic health survey, the maternal mortality ratio was 412 maternal deaths per 100,000 live births for the 7 years before the survey which is still far from the sustainable development goal target of 2030 [4].

Among the 435 women who died after experiencing direct obstetric complications, maternal hemorrhage (23.9%) was the leading cause. The direct obstetric case fatality rate was highest in postpartum hemorrhage followed by uterine rupture [5]. Among the causes of maternal near misses, obstetric hemorrhage was the second most common cause of admissions [6]. 5.5% of facilities had submitted at least one maternal death summary report to the National Maternal Death Surveillance and Response Database. Postpartum hemorrhage followed by severe preeclampsia/eclampsia is the leading primary cause of maternal death [7, 8]. NASG utilization is important while patient referral to higher institutions where definitive management is carried out [9]. A reduction in extreme maternal adverse outcomes and faster recovery from shock are more likely to occur with earlier NASG intervention [1]. The median blood loss decreased by half when the NASG was used for obstetric hemorrhage management, which was associated with significantly reduced maternal mortality among the most severe cases [10]. Postpartum hemorrhage followed by ruptured tubal ectopic pregnancy was the most common indication for NASG use. The shock index after obstetric hemorrhage significantly improved after NASG was applied [11]. NASG intervention has several positive effects, such as decreased maternal mortality and mortality, rapid restoration of vital signs and decreased blood loss [12]. The median recovery time of women from postpartum hemorrhage is shorter when NASG is used while managing the patient [13]. The International Federation of Gynecology and Obstetrics (FIGO) [14] and World Health Organization recommend the use of NASG simultaneously with other treatment alternatives while referral of the patient to the hospital, where definitive treatment is given, significantly reduces maternal morbidity and mortality [15].

The level of NASG utilization varies considerably across countries. For instance, the utilization level ranges from 22.7% to 73.7% in Nigeria [16, 17] and 70.8% in Tanzania [18]. The results of primary studies in Ethiopia revealed that the utilization level varies from 30.7% [19] to 64.2% [20]. This indicates the presence of substantial variation in the use of NASG for obstetric hemorrhage management across the country. Therefore, the aim of this systematic review and meta-analysis (SRMA) was to estimate the pooled prevalence of NASG utilization among obstetric care providers and its predictors in Ethiopia at the national level.

### Objective

To estimate the pooled prevalence of NASG utilization and its predictors in Ethiopia.

## Methods

### Study design and setting

This SRMA was conducted on the use of NASG for obstetric hemorrhage among obstetric care providers in Ethiopia. Preferred Reporting Items for Systematic Review and Meta-Analysis (PRISMA) guidelines were followed (Supplementary file 1). The PRISMA protocol consists of checklists that guide the execution and reporting of the SRMA, which increases the transparency and accuracy of reviews in medicine and other fields. Ethiopia is a low-income country located in the Horn of Africa with a projected population of 123.4 million by 2022, 133.5 million by 2032, and 171.8 million by 2050 [21]. For administrative purposes, Ethiopia is divided into 11 regions and 2 city administrations. The regions are further classified into the zones, and zones are divided into districts. Finally, districts are divided into kebeles (the smallest administrative division contains 2000 to 3500 residents).

### Search strategies and sources of information

This SRMA is registered in the PROSPERO database with ID no. CRD42023484351. A SRMA was conducted on the use of NASG for obstetric hemorrhage management among obstetric care providers and its predictors in Ethiopia. This SRMA aimed to gather information from both published and unpublished literature concerning the prevalence and factors associated with the utilization of NASG for managing obstetric hemorrhage among obstetric care providers in Ethiopia. The search was conducted by two authors, encompassing a comprehensive review of both accessible published studies and undisclosed sources of information on this topic. This approach aims to capture a broad spectrum of evidence, including findings from various research avenues, to comprehensively understand the prevalence and predictors of NASG utilization in obstetric care in Ethiopia (ESL and MA). Studies written in English were eligible for inclusion in the review. Appropriate and comprehensive searches of PubMed, MEDLINE, EMBASE, Google Scholar, HINARI, and Scopus were performed since November 2019. Furthermore, relevant articles found in the gray literature available on local shelves and institutional repositories were systematically reviewed. Medical Subject Headings (MeSH) and key terms were developed using the different Boolean operators ‘AND’ and ‘OR’. The following search terms were used: (Prevalence) OR (proportion) OR (Magnitude) AND (non-pneumatic anti shock garment) AND (utilization) AND (obstetric care providers) AND (obstetric hemorrhage management) AND (predictors) OR (determinants) OR (associated factors) AND (Ethiopia).

The electronic literature search was last performed on November 18/2023. Mendeley reference manager software was used to collect and manage the literature as well as to avoid possible duplications.

### Inclusion

This SRMA included articles conducted in all regional states of Ethiopia, reporting the prevalence/proportion of NASG utilization for obstetric hemorrhage management among obstetric care providers and its predictors and associated factors. All observational study designs were eligible for this SRMA. Both published articles and gray literatures published in English were included.

### Exclusion

Primary studies lacking the outcome of interest (NASG utilization for obstetric hemorrhage management among obstetric care givers and its predictors) were excluded from the SRMA. In contrast, studies of poor quality according to the criteria of reviewing articles were excluded.

### Outcome of measurement

This SRMA has comprised of two main outcomes. The primary outcome of this SRMA was the NASG utilization for obstetric hemorrhage management among obstetric care providers, which is whether healthcare professionals use the NASG for the management of obstetric hemorrhage at least once. Five of the studies [19, 22–25] studied the utilization of NASG specifically for post-partum hemorrhage and the other two studies [20, 26] studied the use of NASG for obstetric hemorrhage in general. The secondary outcome was the predictors of NASG utilization for obstetric hemorrhage management. The attitude of the respondents were measured by using 10 attitude measuring questions and finally favorable attitude declared if the respondent scored 50% or more [19, 20, 22–26].

### Data extraction

All the datasets were exported to the Mendeley reference manager and transferred to a Microsoft Excel spreadsheet to remove duplicate data in the review. Two authors (ESL and MA) independently extracted all important data using a standard data extraction format developed according to the Joanna Briggs Institute (JBI) Reviewers’ manual 2014 [27]. Any disagreements between reviewers were resolved by a third author (GY). A consensus in this study was achieved through thorough critical discussion and evaluation of the articles by all independent reviewers. Each article’s key information, including the author’s name, sample size, publication year, study region, study design, prevalence or proportion of NASG utilization, and adjusted odds ratio along with its 95% confidence interval (CI) for factors associated with NASG utilization, was carefully examined. Only articles that met the predetermined criteria were included as data sources for the final analysis. This meticulous approach ensures that the analysis is based on relevant and rigorous studies that align with the study’s objectives and criteria.

### Quality assessment

Once the database results were exported to the Mendeley reference manager and duplicate results were removed, we used the Newcastle–Ottawa Quality Assessment Scale (NOS) adapted for observational studies to assess the quality of each study included in the SRMA (Table 1). The quality assessment scale evaluates the literature in three categories.

A. Selection (4 points)
B. Comparability (2 points) and
C. Outcome (3 points)

**Table 1:** Newcastle–Ottawa Quality Assessment Scale for cross sectional studies used in the systematic review and meta-analysis, utilization of NASG among obstetric care providers for obstetric hemorrhage and its predictors in Ethiopia, 2023

	**Selection (4)**				**Comparability(2)**	**Outcome (3)**		**Total score**
Author name	Representativeness(1)	Sample size(1)	Non respondents (1)	Ascertainment of the exposure risk factor (1)	The subjects in different outcome groups are comparable, based on the study design or analysis. confounding factors are controlled (2)	Assessment of the outcome (2)	Statistical test (1)	
Abreham A et al. [20]	1	1	1	1	1	2	1	**8**
Zerihun A and Fikrte W [22]	1	1	1	1	2	2	1	**9**
Gadisa B et al. [23]	1	1	1	1	1	2	1	**8**
Birhan T et al. [26]	1	1	1	1	2	2	1	**9**
Merkin B et al. [19]	1	1	1	1	2	2	1	**9**
Yordanos G et al. [24]	1	0	1	1	2	2	1	**8**
Wondimnew G et al. [25]	1	1	1	1	1	2	1	**8**

Two authors (ESL and MA) assessed the quality of each study (methodological quality, sample selection, sample size, comparability, outcome, and statistical analysis). In cases of disagreement between the two authors, a third author (GY) was involved and disagreements were resolved by discussion.

### Data processing and analysis

The extracted Microsoft Excel spreadsheet data were imported into STATA software version 17 (STATA Corporation, Texas, USA) for analysis. A random-effects model was used to estimate the pooled prevalence of NASG utilization among obstetric care providers in Ethiopia. The Cochrane Q-test and I^2^ statistics were computed to assess the heterogeneity among the studies included in the SRMA. Accordingly, if the I^2^ is 0–40%, there is mild heterogeneity, 40 to 70% would indicate moderate heterogeneity, and 70 to 100% would indicate considerable heterogeneity [28]. Funnel plots and Egger’s test were used to assess publication bias. A *p* value < 0.05 indicated that there was significant publication bias. A forest plot was used to present the pooled prevalence of NASG utilization for obstetric hemorrhage management among obstetric care providers with 95% CIs.

### Subgroup and sensitivity analyses

In the course of the analysis, subgroup analyses were conducted based on sample size after determining the mean sample size. To identify determinant factors, the pooled AOR was employed and presented in a forest plot format, along with its corresponding 95% CI. This approach allows for a more detailed examination and presentation of the synthesized data, facilitating a clearer understanding of the factors influencing the utilization of NASG for obstetric hemorrhage management among obstetric care providers in Ethiopia.

## Result

### Characteristics of the included studies

**Fig. 1:**
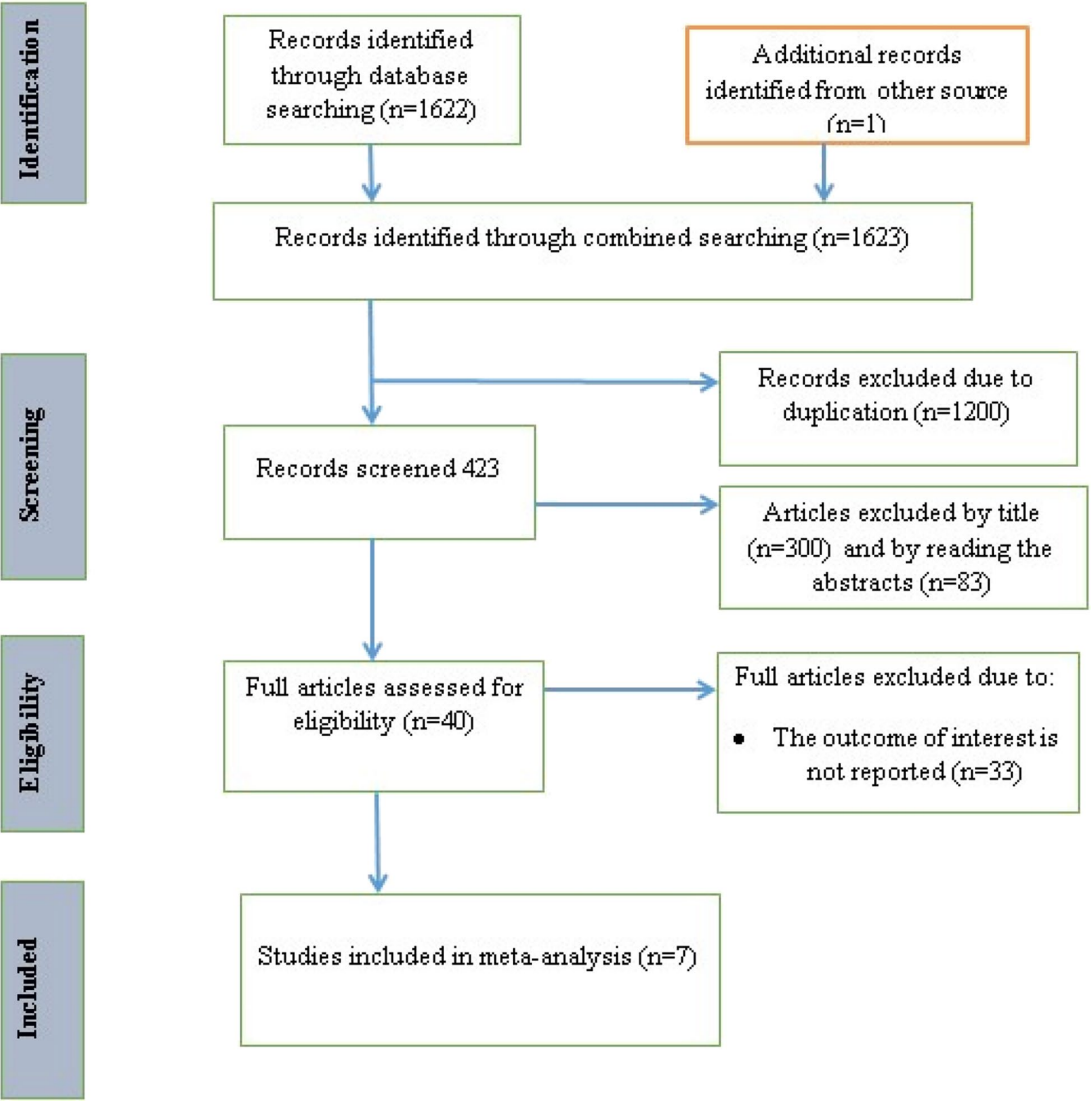
PRISMA flow chart for the selection of a systematic review of NASG utilization for obstetric hemorrhage management among obstetric care providers and predictors in Ethiopia

A total of 1623 articles were found by using our search strategies. After 1200 articles were removed because of duplication, 423 remained. After reviewing their titles and abstracts, 300 articles and 83 articles respectively, were removed. Finally, 40 full-text papers were accessed and evaluated for the predefined inclusion criteria. Thus, 33 more articles were excluded for the aforementioned reasons. Eventually, 7 articles were found to be eligible for inclusion in the final systematic review and meta-analysis (Fig. 1). Among the studies included in this systematic review and meta-analysis, two studies were in Amhara, one in the SNNP region, one in Oromia, one in Addis Ababa, one in Sidama and one in Tigray region. All seven studies were cross sectional. A total of 2335 participants, with the smallest (210) to the largest (412) participants, were included and the prevalence of NASG utilization for obstetric hemorrhage among obstetric care providers in Ethiopia ranged from 30.7 [19] to 64.2% [20]. On the other hand, the quality of each study was assessed by using the Newcastle Ottawa Quality Assessment scale (NOS), and the scores of all included studies ranged between 7 and 9, which indicates good quality (Table 2).

**Table 2:** Characteristics of the included studies in the SRMA of utilization of NASG among obstetric care providers for obstetric hemorrhage and its predictors in Ethiopia, 2023

Seri no	Author	Year	Region	Study design	Sample size	Prevalence of SSI (%)	Quality
1	Abreham A et al. [20]	2022	Tigray	Cross sectional	338	64.2	Good
2	Zerihun A and Fikrte W [22]	2021	Addis Ababa	Cross sectional	388	39.3	Good
3	Gadisa B et al. [23]	2020	Oromia	Cross sectional	220	36.2	Good
4	Birhan T et al. [26]	2023	Amhara	Cross sectional	373	39.4	Good
5	Merkin B et al. [19]	2023	Sidama	Cross sectional	403	30.7	Good
6	Yordanos G et al. [24]	2021	SNNP	Cross sectional	422	48.4	Good
7	Wondimnew G et al. [25]	2023	Amhara	Cross sectional	244	48.08	Good

### Prevalence of NASG utilization in Ethiopia

**Fig. 2:**
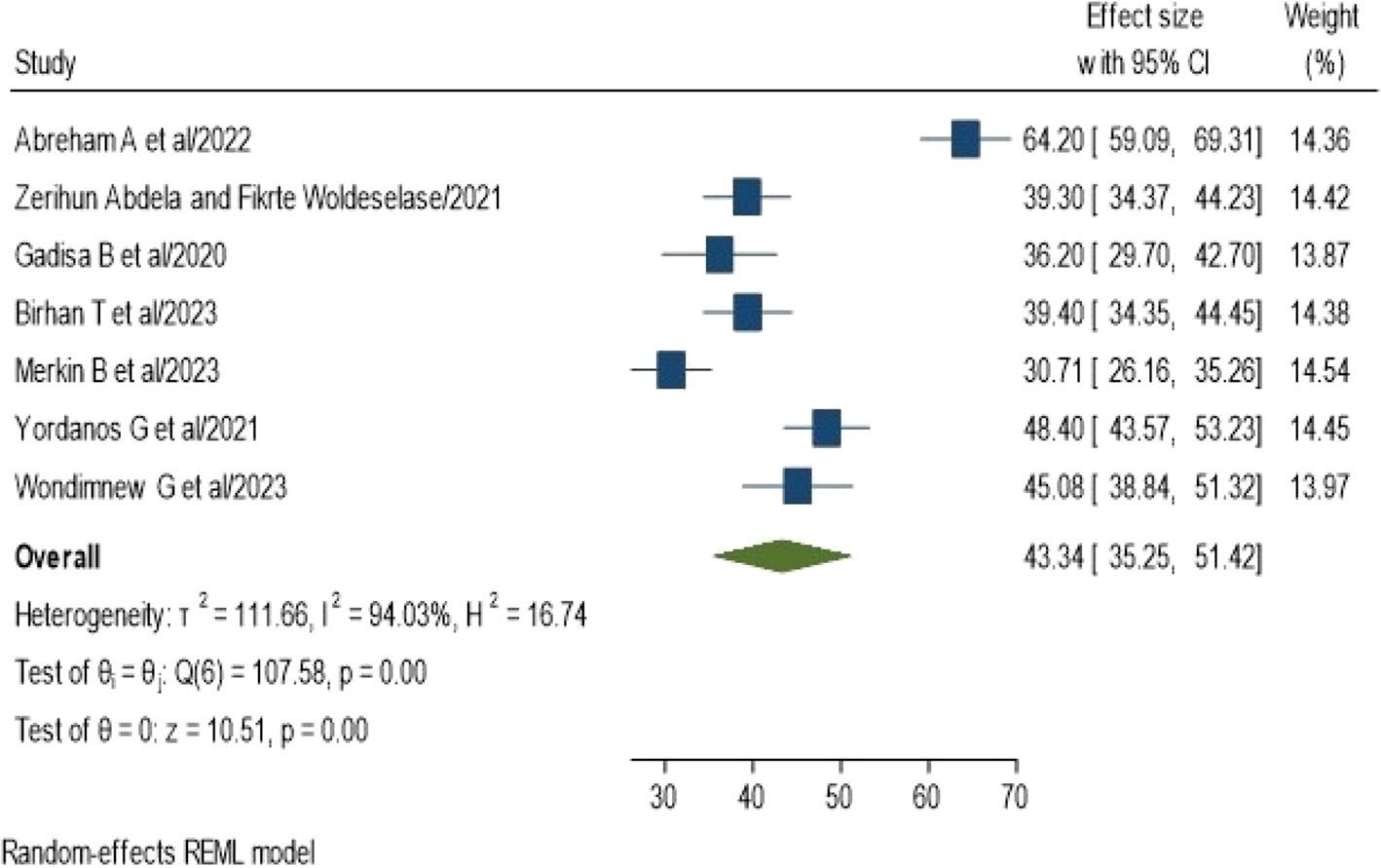
Forest plot showing the pooled prevalence of NASG utilization for obstetric hemorrhage management among obstetric care providers in Ethiopia

The pooled prevalence of NASG utilization for obstetric hemorrhage in Ethiopia was 43.34% (95% CI: 35.25, 51.42%), and the Cochrane heterogeneity index (I^2^ = 94.03%, *P* = 0.00), indicated significant heterogeneity among the studies. Therefore, we used the random effect model to resolve the issue of heterogeneity among the included studies. Moreover, we considered subgroup analysis as a potential way of addressing heterogeneity. The findings are presented in the forest plot (Fig. 2).

### Publication bias

**Fig. 3:**
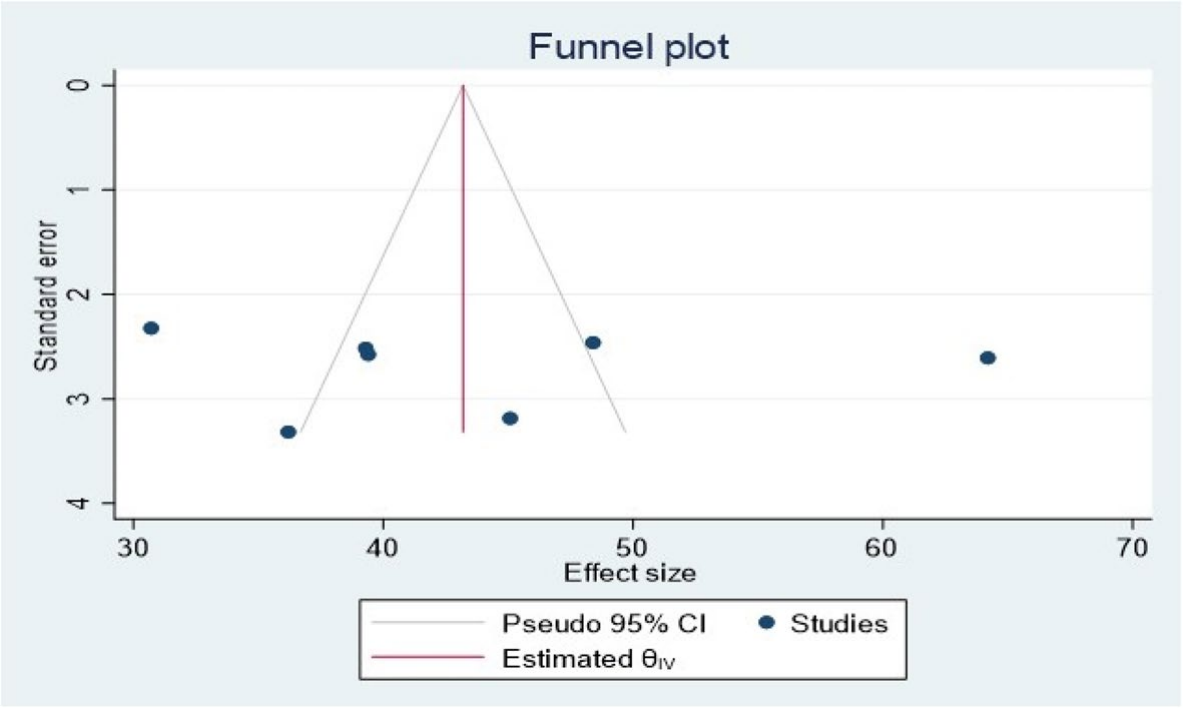
Funnel plot showing the symmetric distribution of each primary study on NASG utilization for obstetric hemorrhage in Ethiopia, 2023

The presence or absence of publication bias was verified by using statistical analysis (funnel plot and Egger’s test (*P* = 0.96 (*P* > 0.05)), and the results showed no publication bias (Fig. 3). Nevertheless, the Egger’s test used to assess the publication bias might be affected by the significant heterogeneity between the included studies and the small number of studies.

### Subgroup analysis of NASG utilization among obstetric care providers in Ethiopia

**Fig. 4:**
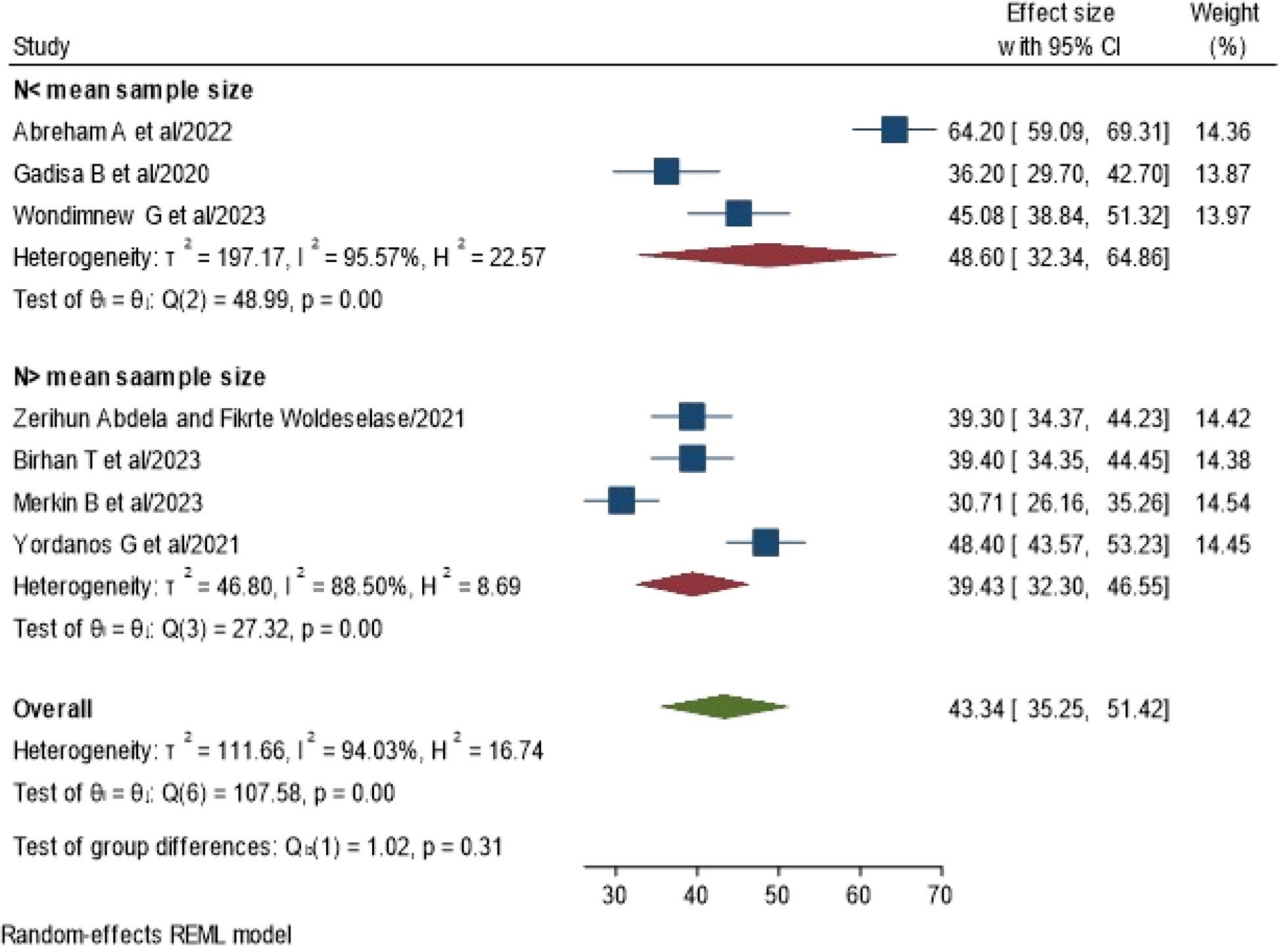
Forest plot showing subgroup analysis of NASG utilization for obstetric hemorrhage management among obstetric care givers in Ethiopia, 2023

In the subgroup analysis, which was conducted based on sample size after computing the mean sample size; 341, the results revealed that the pooled prevalence of NASG utilization for obstetric hemorrhage was greater in studies with a sample size below the mean sample size (48.6%, 95% CI: 32.34, 64.86%, I^2^ = 95.57%, *P* = 0.00). However, the pooled prevalence of NASG utilization for obstetric hemorrhage among obstetric care providers in studies with sample sizes greater than the mean sample size was lower (39.43%; 95% CI: 32.30, 46.55%, I^2^ = 88.5%, *P* = 0.00) (Fig. 4). This suggests that there may be a correlation between sample size and the reported prevalence of NASG utilization, with smaller sample sizes tending to show a higher prevalence. It is important to interpret these findings in the context of potential variations and characteristics associated with studies of different sample size.

### Sensitivity analysis (leave-one-out meta-analysis)

**Fig. 5:**
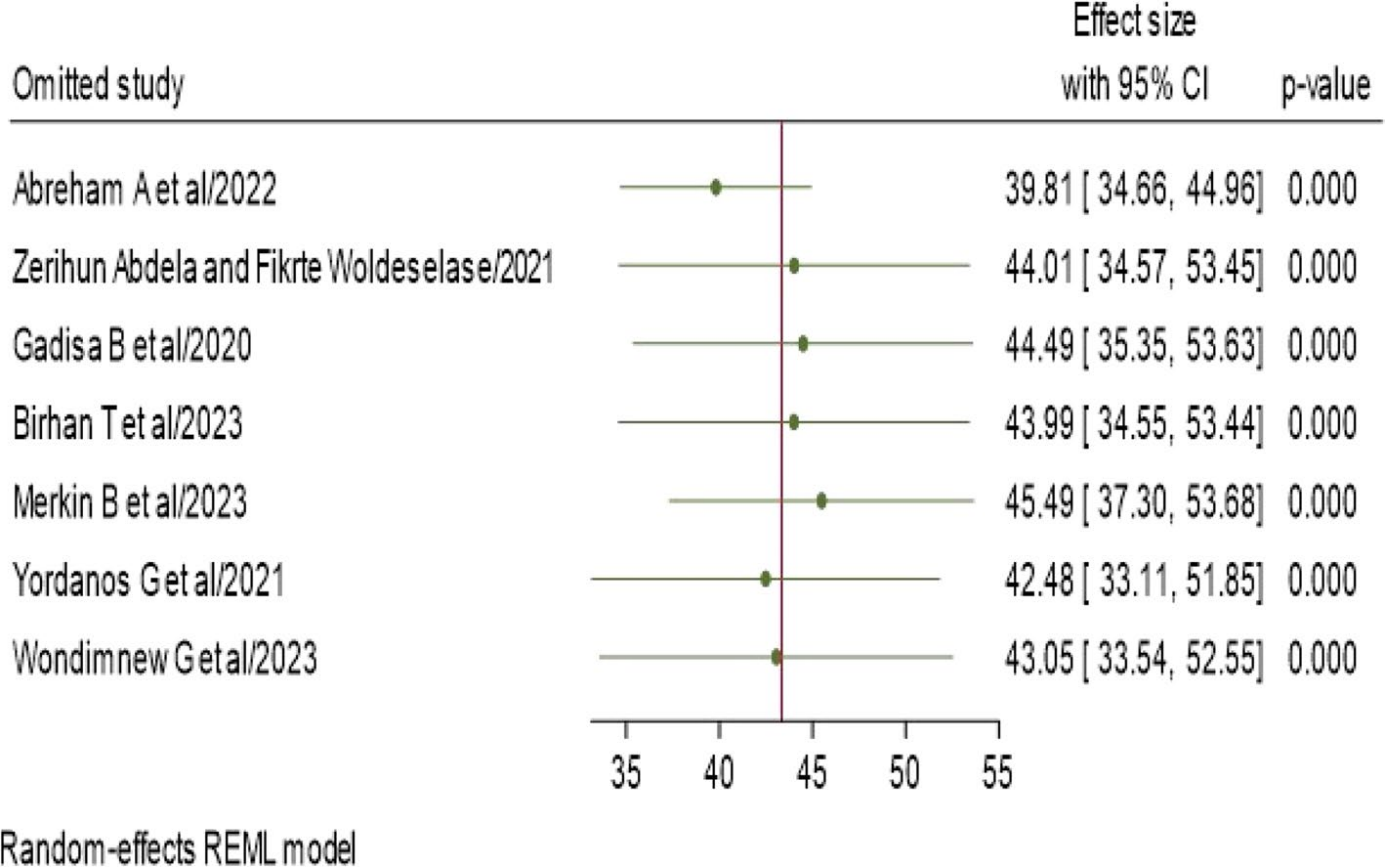
Sensitivity analysis of NASG utilization for obstetric hemorrhage among obstetric care givers in Ethiopia, 2023

This SRMA utilized a random-effects model, and the findings indicated that no individual study significantly influenced the pooled prevalence of NASG utilization for obstetric hemorrhage among obstetric care providers. This suggests that the overall prevalence estimate was robust and not disproportionately influenced by any single study, supporting the reliability and generalizability of the pooled results across the included studies (Fig. 5).

### Determinants of NASG utilization for obstetric hemorrhage in Ethiopia

In this SRMA, variables used by the primary studies were included to determine the predictors of NASG utilization for obstetric hemorrhage. Hence, obstetric care providers who have received training on NASG utilization, who have good knowledge, who have positive attitude and availability of NASG in the working facility care providers are the determinants of NASG utilization for obstetric hemorrhage in Ethiopia. Compared to their counterparts, obstetric care providers who had received training were 3.88 times more likely to use NASG for the management of obstetric hemorrhage (AOR = 3.88, 95% CI: (2.08–5.37)). The likelihood of utilizing the NASG for obstetric hemorrhage was two times greater for obstetric care providers who had good knowledge towards NASG than their counterparts (AOR = 1.99, 95% CI: (1.28–3.16)). The attitude of health care providers also matters in the utilization of NASG. Obstetric care providers with positive attitude have used the device for obstetric hemorrhage management about 2.14 times more than those with negative attitude (AOR = 2.14, 95% CI: (1.62–2.75). The presence or absence of the device in health facilities also determines its utilization by obstetric care providers. They are five times more likely to utilize the device for obstetric hemorrhage if it is available in the hospital or health center they are working than their counterparts (AOR = 4.89. 95% CI: (2.88–8.32)) (Table 3).

**Table 3:** Factors associated with utilization of NASG for obstetric hemorrhage management among obstetric care providers in Ethiopia

Variables	Author name/publication year	AOR	95% CI	Pooled AOR	95%CI
Training	Abreham A et al./2022 [20]	1.43	0.62–3.12	**3.88**	**2.08–5.37**
	Zerihun Abdela and Fikrte Woldeselase/2021 [22]	5.469	1.08–24.75		
	Gadisa B et al./2020 [23]	13.15	4.81–36.0		
	Birhan T et al./2023 [26]	3.3	1.46–7.48		
	Merkin B et al./2023 [19]	4.18	2.16–8.07		
	Yordanos G et al./2021 [24]	2.9	1.7–4.2		
	Wondimnew G et al./2023 [25]	2.2	1.1–4.4		
Knowledge	Abreham A et al./2022 [20]	1.06	0.56–1.54	**1.99**	**1.28–3.16**
	Zerihun Abdela and Fikrte Woldeselase/2021 [22]	1.353	0.53–3.48		
	Gadisa B et al./2020 [23]	3.96	1.67–9.40		
	Birhan T et al./2023 [26]	1.11	0.62–1.98		
	Merkin B et al./2023 [19]	2.5	1.26–4.98		
	Yordanos G et al./2021 [24]	2	1.7–3.4		
	Wondimnew G et al./2023 [25]	5.2	2.5–10.7		
Attitude	Abreham A et al./2022 [20]	2.07	0.85–5.03	**2.14**	**1.62–2.75**
	Zerihun Abdela and Fikrte Woldeselase/2021 [22]	1.47	0.34–6.25		
	Gadisa B et al./2020 [23]	3.54	1.37–9.13		
	Birhan T et al./2023 [26]	1.63	1.14–2.84		
	Merkin B et al./2023 [19]	2.38	1.18–4.76		
	Yordanos G et al./2021 [24]	2.5	1.42–5		
	Wondimnew G et al./2023 [25]	2.5	1.1–5.7		
Availability of NASG	Abreham A et al./2022 [20]	3.66	0.66–20.27	**4.89**	**2.88–8.32**
	Zerihun Abdela and Fikrte Woldeselase/2021 [22]	4.71	2.17–10.26		
	Birhan T et al./2023 [26]	9.17	5.1–16.46		
	Merkin B et al./2023 [19]	4.6	1.6–13.24		
	Wondimnew G et al./2023 [25]	2.7	1.3–5.5		

## Discussion

Obstetric hemorrhage is one of the leading causes of maternal mortality and severe morbidities. Especially in resource limited areas, where definitive management is not easily available, the use of this first aid device has significant role in reducing the burden of the problem. This SRMA was conducted to determine the pooled prevalence of NASG utilization for obstetric hemorrhage and its predictors among obstetric care providers in Ethiopia. The result of this SRMA showed that the overall rate of NASG utilization for obstetric hemorrhage among obstetric care providers was 43.34% (95% CI: 35.25, 51.42%). This study is in line with studies conducted in Nigeria [17] and Bayelsa State, Nigeria [29], which revealed that 36.4% and 46.4% of obstetric care providers respectively, utilize the NASG for obstetric hemorrhage management. However, this study showed that the utilization of NASG for obstetric hemorrhage management is much lower than reported in studies conducted in Ibadan, Nigeria [30], and rural Tanzania [18] which revealed that 73.7% and 70.8% of obstetric care providers had utilized the device while managing obstetric hemorrhage respectively. This could be due to the variation in socio demographic characteristics of the study settings and there might also be differences in the training, availability of the device in the facility and in the supervisions. Furthermore, the study findings in rural Tanzania were from an ongoing project of NASG implementation, which was supported by simulation training, repeated training and supportive supervisions, increases the level of NASG utilization. On the other hand, the result of this SRMA revealed that the utilization level of NASG is much greater than a study conducted in Ogun state, Nigeria [16] which indicates that only 22.7% of obstetric care providers utilize the device while managing obstetric hemorrhage.

In addition, obstetric care providers who had received training on NASG, having good knowledge, having a positive attitude and availability of the device in the facility were significantly associated with the utilization of NASG for obstetric hemorrhage management in Ethiopia. One of the determining factors of NASG utilization is receiving training, which revealed that obstetric care providers who received training on NASG use are 3.88 times more likely to utilize the device for obstetric hemorrhage management than their counterparts. This is consistent with studies conducted in rural Tanzania [18]. A possible explanation for this association is that attending training helps obstetric care providers improve the basic knowledge and skills of NASG application and eventually increases the utilization level of the device for obstetric hemorrhage.

The association between NASG utilization and knowledge of the obstetric care providers were also tested. The result showed that having good knowledge of NASG use increases the likelihood of NASG utilization while managing obstetric hemorrhage by 1.99 times more than their counterparts. This is because having good knowledge enhances the promptness and certainty of obstetric care providers toward NASG utilization while managing obstetric hemorrhage. This study is supported by studies conducted in Ogun state [17]. However, there was no association between knowledge and utilization of the device in a study conducted in Ibadan, Nigeria [30]. This could be due to the small sample size they used, which included 140 obstetric care providers.

The utilization of NASG for obstetric hemorrhage management was also determined by the attitudes of obstetric care providers towards NASG use. Thus, the likelihood of utilizing NASG for obstetric hemorrhage management among obstetric care providers with a positive attitude is 2.16 times greater than their counterparts. This may be explained by the fact that most of inspiring factors for obstetric care providers’ NASG utilization are a result of positive attitudes. On the other hand, the positive attitude of obstetric care providers is basically driven by their belief in the effectiveness and safety of NASG for managing obstetric hemorrhage. This finding is consistent with a study conducted at Adeoyo maternity teaching hospital, Ibadan [30]. However, a study conducted in selected hospitals in Ondo state [31], revealed the absence of association between NASG utilization and the attitude of obstetric care providers.

The likelihood of utilizing NASG for obstetric hemorrhage management among obstetric care providers working in a facility where NASG is available is 4.89 times greater than those who work in facilities, where NASG is not available. This result is supported by a study conducted in Nigeria [32], specialist hospital Sokoto [33] and Ibadan, Nigeria [30]. The possible explanation might be due to availing the device in the facility shows the commitment to utilize the device for obstetric hemorrhage management. Furthermore, the recommendation of WHO and FIGO as an element of obstetric hemorrhage management motivates the obstetric care providers to utilize the device in hand. The findings of SRMA underscore the urgency of implementing comprehensive interventions to enhance the knowledge and attitude of obstetric care providers. Continuous training initiatives and supportive measures are crucial components of these interventions. It is imperative that all stakeholders engage proactively in training efforts and ensure the widespread availability of NASG in healthcare facilities. This concerted approach is essential for improving the utilization of NASG in the management of obstetric hemorrhage. Furthermore, there is a clear call for scholars to delve into investigating the potential facilitators and challenges associated with NASG use. Incorporating a qualitative approach in future research endeavors is expected to yield valuable insights that can contribute to a more nuanced understanding of NASG utilization. Despite the efforts to minimize limitations in this SRMA, it is important to acknowledge certain constraints. The inclusion of only seven studies identified by the search strategy and the necessity to compare findings with primary studies due to the absence of similar reviews are acknowledged limitations. Moreover, the power of Egger’s test for publication bias may be compromised when the number of included primary studies is low, especially in the presence of significant heterogeneity among the studies. These limitations should be considered when interpreting the results of this SRMA.

## Conclusion

The pooled prevalence of NASG utilization for managing obstetric hemorrhage among obstetric care providers was low. This emphasizes the imperative need for meticulously crafted intervention plans aimed at increasing the utilization of NASG. Such interventions are essential not only for saving the lives of women but also for preventing potential short- and long-term disabilities. The study identified key factors associated with the use of NASG, including receiving training, good knowledge, positive attitudes, and the availability of the device in healthcare facilities. The results highlight the significance of enhancing the knowledge and attitude of obstetric care providers through continuous training and support. Furthermore, policymakers and stakeholders are urged to prioritize the widespread availability of the NASG devices in all health facilities. This concerted effort is crucial for promoting and improving the use of NASG in the management of obstetric hemorrhage.

### Supplementary Information

Supplementary Material 1.

## Data Availability

No datasets were generated or analysed during the current study.

## Abbreviations

AOR: Adjusted Odds Ratio
CI: Confidence Interval
NASG: Non pneumatic anti-shock garment
SNNP: South nation nationalities and people
SRMA: Systematic Review and Meta-Analysis
